# Molecular Biologic Approach to the Diagnosis of Pancreatic Carcinoma Using Specimens Obtained by EUS-Guided Fine Needle Aspiration

**DOI:** 10.1155/2012/243524

**Published:** 2012-11-08

**Authors:** Kiyohito Kato, Hideki Kamada, Takayuki Fujimori, Yuuichi Aritomo, Masahiro Ono, Tsutomu Masaki

**Affiliations:** Department of Gastroenterology and Neurology, Faculty of Medicine, Kagawa University, 1750-1 Ikenobe, Miki-cho, Kita-gun, Kagawa Prefecture, Takamatsu 761-0793, Japan

## Abstract

We review the utility of endoscopic ultrasound-guided fine needle aspiration (EUS-FNA), a rapid, safe, cost-effective, and accurate diagnostic modality for evaluating pancreatic tumors. EUS-FNA is currently used for the diagnosis and staging of pancreatic tumors. The sensitivity of EUS-FNA for pancreatic malignancy ranges from 75% to 94%, and its specificity approaches 100% in most studies. However, EUS-FNA has some limitations in the diagnosis of well-differentiated or early-stage cancers. Recent evidence suggests that molecular biological analysis using specimens obtained by EUS-FNA improves diagnostic sensitivity and specificity, especially in borderline cytological cases. It was also reported that additional information regarding patient response to chemotherapy, surgical resectability, time to metastasis, and overall survival was acquired from the genetic analysis of specimens obtained by EUS-FNA. Other studies have revealed that the analysis of KRAS, MUC, p53, p16, S100P, SMAD4, and microRNAs is helpful in making the diagnosis of pancreatic carcinoma. In this paper, we describe the present state of genetic diagnostic techniques for use with EUS-FNA samples in pancreatic diseases. We also discuss the role of molecular biological analyses for the diagnosis of pancreatic carcinoma.

## 1. Introduction

Pancreatic cancer is now the fifth-leading cause of cancer-related death in Japan, and the annual mortality due to pancreatic cancer is estimated to be over 20,000 individuals. The 5-year survival rate of pancreatic cancer is as low as 5.5%, and the poor prognosis is attributed to the difficulty in detection of the disease at an early stage due to its high malignancy potential, the propensity of the cancer to metastasize, and the cancer's high resistance level to antitumor agents.

Endoscopic ultrasound-guided fine needle aspiration (EUS-FNA) was introduced into clinical practice in the early 1990s, and it is now considered one of the most useful methods for histological diagnosis and staging of pancreatic cancers [1, 2]. EUS-FNA of the pancreas is an efficient and minimally invasive procedure for the diagnosis and staging of pancreatic cancer. Various studies conducted since 2003 have found that EUS-FNA for pancreatic solid masses showed the following values: sensitivity, 78%–95%; specificity, 75%–100%; positive predictive value, 98%–100%; negative predictive value, 46%–80%; accuracy, 78%–95% [3]. However, specimens obtained by EUS-FNA are tiny and fragmented so that a definitive diagnosis is frequently challenging for pathologists. Nevertheless, it is important to determine the histological subtype of the tumor, especially in an unresectable tumor because the choice of treatment largely depends on the subtype [1]. It is thus, necessary to improve the diagnostic sensitivity and specificity of EUS-FNA. In this paper, we describe the current state of the genetic diagnosis techniques, and we discuss the role of molecular biological analyses for the diagnosis of pancreatic carcinoma.

## 2. Oncogene

### 2.1. KRAS (Kirsten Rat Sarcoma-2 Virus)

The KRAS oncogene is frequently mutated in human malignancies such as colon, lung, and ovarian cancer. In pancreatic cancer, mutations in KRAS are found in more than 90% of patient samples. The most frequent mutation is the constitutively active KRAS^G12D^ allele. Interestingly, KRAS mutations are frequently detected in the most common precursor lesion to pancreatic cancer, pancreatic intraepithelial neoplasia (PanIN), indicating a potential role for early pancreatic cancer in the disease [4, 5].

Several research groups have suggested that the presence of KRAS gene mutations in tissue obtained by EUS-FNA improved the accuracy of the diagnosis of pancreatic cancer [3, 6–8]. Wang et al. [9] evaluated the usefulness of a novel method including EUS-FNA for the detection of mutations in the KRAS gene for the diagnosis of pancreatic cancer. They evaluated biopsies histopathologically and cytopathologically. In the pancreatic cancer cases studied, 88.9% (48/54; 95% confidence interval [CI]: 80.5%–97.2%) had KRAS gene mutations (codons 12 and 13), whereas 61.1% (33/54; 95% CI: 48.1%–74.1%) were unequivocally diagnosed by histocytopathology. Those authors also reported that compared with the measurement of serum CA19-9, the sensitivity of detection by KRAS mutations (76.2%) and the sensitivity of detection by the combination of KRAS mutations and serum CA19-9 (81%) were significantly higher than that for serum CA19-9 alone (52.4%). A logistic regression model showed that the KRAS mutation was significant (odds ratio  =  5.830; CI: 1.531–22.199, *P* = 0.01), but not serum CA19-9.

Bournet et al. reported that codon-12 KRAS point mutation was found in 66% of pancreatic adenocarcinoma samples obtained by EUS-FNA [10]. No case of chronic pancreatitis displayed KRAS mutation. In that study, the following values were obtained for cytopathology alone for the diagnosis of pancreatic adenocarcinoma versus chronic pancreatitis: sensitivity, 83%; specificity, 100%; positive predictive value, 100%; negative predictive value, 56%; overall accuracy, 86%. When the KRAS mutation analysis was combined with cytopathology, these values reached 88%, 100%, 100%, 63%, and 90%, respectively. Bournet et al. also noted that the KRAS analysis in addition to EUS-FNA biopsy was useful in strongly suggesting a benign lesion, when chronic pancreatitis presented as a pseudotumor a negative finding (wild-type KRAS). Reicher et al. demonstrated that combining routine cytology with fluorescence in situ hybridization (FISH) and KRAS analyses improves the diagnostic yield from EUS-FNA of solid pancreatic masses [11].

Takahashi et al. reported that EUS-FNA with the addition of KRAS mutation analysis to the cytopathologic and histopathologic analysis was highly accurate for the differentiation of benign versus malignant pancreatic mass lesions [7]. In the 62 pancreatic cancer cases examined in the present study, with respect to cytopathologic diagnosis, the KRAS point mutation was found in 50% (2/4) of the cases with a result of no malignancy, in 71% (5/7) of the cases in which malignancy was suspected, and in 76% (39/51) of the cases in which malignancy was diagnosed (Table 1). With respect to histopathologic diagnosis (Table 2), the KRAS point mutation was detected in 43% (3/7) of the cases with a result of insufficient material, in 64% (7/11) of those with no malignancy, in 80% (4/5) of the cases with a finding of atypia, in 80% (12/15) of the cases in which malignancy was suspected, and in 83% (20/24) of the cases in which malignancy was diagnosed. These studies indicate that samples obtained by EUS-FNA analyses of KRAS mutation improve the diagnosis of pancreatic cancer. In addition, such an analysis is more useful than combining conventional methods.

## 3. Tumor Suppressor Genes

### 3.1. p53

Inactivation of the p53 tumor suppressor gene is very common in almost all human cancers [12]. Normal p53 protein functions in cell-cycle regulation, in the maintenance of genomic stability, and in controlled cell death (apoptosis). A mutated p53 protein is capable of inactivating the normal function of p53 in cells, even in the presence of the normal (wild-type) protein. Most inactivating mutations in p53 consist of single point mutations in evolutionarily conserved domains that change the amino acid composition of the resulting p53 protein. The majority of inactivating mutations in p53 leads to an increased stability of the p53 protein. Under normal conditions, p53 protein levels in the cell nucleus are not detectable by standard protein immunohistochemistry, but in cells with mutated p53, the accumulation of p53 protein is easily detectable. Inactivation of the p53 tumor suppressor gene is common in pancreatic carcinoma and is found in 50%–70% of cases [13–15].

Itoi et al. conducted a p53 immunohistochemical analysis in FNA biopsy specimens obtained from chronic pancreatitis and pancreatic cancers [16]. They reported that p53 protein overexpression was observed in 67% of the samples with pancreatic cancer, but not in samples with chronic pancreatitis, and they found that by using the combination of p53 protein overexpression and conventional histological examination, the diagnosis of pancreatic cancers improved as follows: 90% sensitivity, 91% specificity, and 92% accuracy, whereas the conventional histological EUS-FNA diagnostic test statistics for the pancreatic masses were as follows: 76% sensitivity, 91% specificity, and 79% accuracy.

Jahng et al. reported that the combination of p53 and cytology to detect malignancy increased the sensitivity to 51% with 100% specificity, whereas cytology alone had 41% sensitivity and 100% specificity [17].

### 3.2. p16 (CDKN2A, INK4)

The gene encoding the cell-cycle regulatory protein p16 is localized on chromosome band 9p21. Mutations in the p16 gene are associated with an increased risk of a wide range of cancers, and alterations of the gene are frequently seen in cancer cell lines. Pancreatic adenocarcinoma is often associated with mutations in the p16 gene [18, 19].

p16 mutations in EUS-FNA specimens revealed sensitivity and specificity of 13% and 100%, respectively, for a pancreatic cancer diagnosis. However, when detection by monitoring the loss of heterozygosity (LOH) was used, the sensitivity was improved to 85% for allelic losses at 9p [20].

### 3.3. SMAD4 (DPC4)

SMAD4 is often found mutated in many cancers. It acts as a tumor suppressor that functions in the regulation of the TGF-*β* signal transduction pathway, which negatively regulates the growth of epithelial cells and the extracellular matrix (ECM). SMAD4 is inactivated in approximately 55% of pancreatic cancers, either by homozygous deletion (30%) or by intragenic mutations and loss of second allele (25%) [21].

LOH on 18q with SMAD4 is detected in 43% of EUS-FNA specimens from chronic pancreatitis and in 78% of EUS-FNA specimens from pancreatic cancer [20]. In the present study, using the LOH test for pancreatic cancer diagnosis at chromosomal position 18q with SMAD4, the sensitivity and specificity of the pancreatic cancer were 78% and 57%, respectively.

## 4. Glycosylated Proteins

### 4.1. MUCs

Mucins (MUCs) are heavily glycosylated high molecular weight glycoproteins with an aberrant expression profile in various malignancies [22]. It is reported that distinct gene MUC genes have been identified at least 14 genes. Under normal circumstances, mucins are known to play a protective role for epithelial tissues. Alterations in the expression and in the structure of mucins have been reported in both preneoplastic and neoplastic lesions [23, 24]. In tissue specimens of pancreatic cancer, overexpression of MUC1 (membrane-bound pan-epithelial mucin) and MUC6 (gastric pyloric gland-type secretory mucin) and the de novo expression of MUC5AC (gastric surface secretory-type mucin) have been observed as early events in pancreatic carcinogenesis in all stages of PanIN and invasive ductal adenocarcinomas, whereas goblet cell metaplasia with associated MUC2 (intestinal-type secretory mucin) expression was an extremely rare event in most of the studies.

Giorgadze et al. examined the epithelial expression profiles of MUC1, −2, −5AC, and −6 on cell block sections of EUS-FNA samples [25]. They observed the expression of MUC1 and −6 but not that of MUC2 or −5AC in nonneoplastic pancreatic samples. MUC5AC expression in differentiating ductal adenocarcinomas from benign conditions demonstrated better operating characteristics than either MUC1 or MUC6. Those authors used a panel of three antibodies, and the combination of MUC1+/MUC2−/MUC5AC+ was noted in 70.0% of the ductal carcinoma samples.

Wang et al. immunohistochemically investigated the expression of mucins (MUC1, MUC2, and MUC5AC) in EUS-FNA samples of pancreatic occupying lesions [26]: the prevalence of MUC1, MUC2, and MUC5AC expression in pancreatic cancers were 77.5% (31/40), 10.0% (4/40), and 80.0% (32/40), respectively, and in the benign pancreatic diseases the corresponding values were 25% (4/16), 31.3% (5/16), and 43.8% (7/16). As shown in Table 3, they investigated whether the combination of MUC1+cytology and MUC5AC+cytology could provide higher sensitivity and accuracy in a pancreatic cancer diagnosis in comparison with only a cytologic diagnosis. Carrara et al. found that the prevalence of MUC7 in ductal adenocarcinoma was 73.0% [27]; MUC7 expression was highly significant for adenocarcinoma (*P* = 0.007) and borderline for intraductal papillary mucinous neoplasm (IPMN) (*P* = 0.05). MUC7 was expressed in 37.5% of the chronic pancreatitis cases examined.

The MUC expression profile in EUS-FNA biopsy specimens has high value for the diagnosis of pancreatic cancer and mucinous neoplasms. It can play an important role in the clinical diagnosis of pancreatic occupying lesions.

## 5. Calcium Binding Protein

### 5.1. S100P

A member of the S100 family of calcium-binding proteins. S100P is a 95-amino-acid protein [28]. S100P expression is described in many different cancers, and its expression is associated with drug resistance, metastasis, and poor clinical outcome. S100P has been shown to mediate tumor growth, drug resistance, and metastasis through RAGE (receptor for advanced glycation end products) [29]. Several studies have documented that S100P is highly overexpressed in pancreatic ductal adenocarcinoma [30–33]. Several studies examined the usefulness of S100P in the diagnosis of pancreatic adenocarcinoma using EUS-FNA [34–36]. Deng et al. [36] indicated that S100P has 100% sensitivity and 92.8% specificity and a diagnostic accuracy of 100% in six atypical and suspicious cases histologically proven to be pancreatic adenocarcinoma. Daniel et al. reported that S100P had 90% sensitivity and 67% specificity or diagnosing pancreatic adenocarcinoma in cytological specimens obtained by EUS-FNA. They also studied other proteins overexpressed in pancreatic adenocarcinoma (i.e., prostate stem cell antigen, fascin, 14-3-3 sigma, and mesothelin), and they confirmed that S100P was the best marker to diagnose pancreatic adenocarcinoma. These results suggest that the use of S100P in the molecular diagnosis of pancreatic adenocarcinoma using EUS-FNA can increase the diagnostic accuracy for pancreatic cancer.

## 6. MicroRNAs (miRNAs)

MicroRNAs (miRNAs) are small, noncoding RNA molecules of 17 to 27 nucleotides in length. miRNAs play critical roles in diverse biological processes such as cell development and differentiation and the control of cellular proliferation. MiRNAs are aberrantly expressed in virtually all human cancer types, and it has been reported that miRNAs may function as tumor suppressors or oncogenes, and that alteration in miRNA expression may play a critical role in tumorigenesis and cancer progression. In our recent study, we showed that various miRNAs are changed in gastric cancer cells by application of the antidiabetic drug metformin [37]. Preis et al. used an optical intensity analysis to investigate miRNA expression in cytokeratin 19 (CK19)-positive epithelial cells in surgically resected pancreatic cancer tissues and EUS-FNA samples [38]. In their study, the expression levels of miR-10b were increased in the pancreatic cancer cells in the EUS-FNA samples compared to the levels in pancreatic ductal cells in benign lesions. They also found that lower levels of miR-10b in the cancer cells were associated with improved response to neoadjuvant gemcitabine-based chemoradiotherapy, surgical resectability, time to metastasis, and overall survival.

However, miRNAs are a relatively new focus of molecular biological analyses in pancreatic cancer using EUS-FNA specimens. miRNAs may eventually be useful factors in the diagnosis of pancreatic cancer, but further studies are needed.

## 7. Molecular Diagnosis by EUS-FNA

Jones et al. reported a comprehensive genetic analysis of 24 pancreatic cancers in 2008 [39]. They found that pancreatic cancers contain an average of 63 genetic alterations, the majority of which are point mutations. These alterations defined a core set of 12 cellular signaling pathways and processes (including KRAS, TGF-*β* signaling and p16) that were each genetically altered in 67% to 100% of the tumors. These findings are useful for the development of not only treatments for pancreatic carcinoma, but also diagnostic approaches to this cancer. With the above described genes, the diagnosis rate for pancreatic cancer using EUS-FNA may be improved.

Many genes are reported to be related to pancreatic cancer, but there are few reports about the genes used in the molecular diagnosis by EUS-FNA. The GNAS gene encodes the *α*-subunit of the stimulatory G-protein (G*α*s), which mediates the regulation of adenylate cyclase activity through G-protein-coupled receptors. Activating mutations of GNAS are reportedly prevalent in IPMN [40]. GNAS is mutated in approximately 60% of IPMNs and in some invasive pancreatic cancers arising in association with an IPMN [41].

BRAF is a member of the Raf kinase family of serine/threonine-specific protein kinases. This protein plays a role in regulating the MAP kinase/ERKs signaling pathway, which affects cell division, differentiation, and secretion. The BRAF gene is activated by oncogenic RAS, leading to cooperative effects in cells responding to growth factor signals. Schönleben et al. evaluated mutations of BRAF in 36 IPMN/IPMC (intraductal papillary mucinous carcinoma) samples by direct genomic sequencing [42]. Exons 5, 11, and 15 for BRAF were examined. One missense mutation (2.7%) was identified within exon 15 of BRAF. The mutations appear to be somatic, since the same alterations were not detected in the corresponding normal tissues. Schönleben et al. argued that oncogenic properties of BRAF contribute to the tumorigenesis of IPMN/IPMC. The diagnosis of pancreatic cancer by EUS-FNA using these genes merits further studies.

## 8. Conclusions

EUS-FNA is a rapid, safe, cost-effective and accurate diagnostic modality for evaluating pancreatic tumors. The sensitivity of EUS-FNA for pancreatic malignancy ranges from 75% to 94%, and its specificity approaches 100% in most studies [35, 43, 44]. However, EUS-FNA has some limitations in distinguishing between well-differentiated adenocarcinoma and reactive changes, because of overlapping cytological features between neoplastic and reactive ductal epithelium. Therefore, false-negative rates and atypical or suspicious diagnoses remain relatively high.

Based on this paper, a diagnostic algorithm reflecting the most efficient approach to distinguish pancreatic cancer from benign lesions in EUS-FNA samples can be constructed. Since EUS-FNA still has the highest diagnostic relevance, reaching 100% predictive values while showing acceptable sensitivity and specificity, it should remain a preferred method for the examination of a focal pancreatic mass. Only a subset of EUS-FNA inconclusive samples should be further examined by genetic analyses, and the positive cases from molecular diagnoses using the various markers should proceed to treatment. Future studies of EUS-FNA and genetic analyses can be expected to obtain additional information toward improving response to chemotherapy, surgical resectability, time to metastasis, and overall survival in pancreatic cancer cases [45–47].

As shown in Table 4, summarized reports of diagnosis of pancreatic cancer by genetic analyses, our paper indicates that genetic diagnosis could be useful to improve the diagnostic sensitivity and specificity of EUS-FNA, especially in those borderline cases that cannot be rendered with certainty by morphology alone.

## Figures and Tables

**Table 1:** Relationship between cytopathologic evaluation and KRAS point mutation in specimens of pancreatic cancer (62 cases) obtained by EUS-FXA; citation from [7].

	Cytology	KRAS point mutation positive
No malignancy	4	2 (50%)
Suspicion of malignancy	7	5 (71%)
Malignancy	51	39 (76%)

Total	62	46

**Table 2:** Relationship between histopathologic evaluation and KRAS point mutation in specimens of pancreatic cancer (62 cases) obtained by EUS-FXA; citation from [7].

	Histology	KRAS point mutation positive
Insufficient material	7	3 (43%)
No malignancy	11	7 (64%)
Atypical	5	4 (80%)
Suspicion of malignancy	15	12 (80%)
Malignancy material	24	20 (83%)

Total	62	46

**Table 3:** Accuracy of the 3 tests for diagnosing pancreatic cancer; citation from [16].

Diagnosis	Test	Case (*n*)	Sensitivity (%)	Specificity (%)	Accuracy (%)
	Cytology analysis	56	65	93.8	73.2
Pancreatic cancer	Cytology analysis + MUC1(+)	56	85	100	89.3
	Cytology analysis + MUC5AC(+)	56	90	93.8	91.1

**Table 4:** Studies of molecular biologic diagnosis of pancreatic carcinoma using specimens obtained by EUS-FNA.

Biological analyses	Author	Year	EUS-FNA samples	Sensitivity	Specificity	Positive predictive value	Negative positive value	Accuracy	Diagnosis
	Reicher et al. [11]	2011	46	87.9%	93.8%	96.7%	78.9%	89.8%	Combination with cytology and FISH
KRAS	Bournet et al. [10]	2009	178	88.0%	100%	100%	63.0%	90.0%	Combination with cytopathology
	Maluf-Filho et al. [8]	2007	74	90.9%	47.6%	89.5%	98.1%	89.2%	Combination with cytopathology

P53	Jahng et al. [17]	2010	61	51.0%	100%				Combination with cytology
	Itoi et al. [16]	2005	62	90.0%	91.0%			92%	Combination with cytopathology

P16	Salek et al. [20]	2007	101	85.0%	100.0%				Monitoring the loss of heterozygosity
SMAD4	Salek et al. [20]	2007	101	78.0%	57.0%				Monitoring the loss of heterozygosity

MUCs	Wang et al. [26]	2007	40	100%				91.1%	MUC5AC+cytology
	Giorgadze et al. [25]	2006	30	70%	100%	100%	100%	75%	Combination of MUC1+/ MUC2−/ MUC5AC+

S100P	Kosarac et al. [34]	2011	14	78.2%	87.5%				
	Daniel et al. [35]	2011	62	90.0%	67.0%				

*FISH: fluorescense in situ hybridization.
